# Clinical-scale, modular manufacturing of tumor-reactive TILs using a closed and automated culture system

**DOI:** 10.3389/fimmu.2024.1483254

**Published:** 2024-12-09

**Authors:** Christina Völzke, Lisa Ehrhardt, Laura Fischer, Peter Maul, Carina Wenzel, Arina Riabinska, Elvira Criado-Moronati, Mike Dienstbier, Jessica Hassel, Danmei Zhang, John B. Haanen, Rupert Handgretinger, Ian R. Hardy, Bianca Heemskerk, Andrzej Dzionek

**Affiliations:** ^1^ Research and Development, Miltenyi Biotec, Bergisch Gladbach, Germany; ^2^ Heidelberg University, Medical Faculty Heidelberg, Department of Dermatology and National Center for Tumor Diseases (NCT), NCT Heidelberg, a partnership between DKFZ and University Hospital Heidelberg, Heidelberg, Germany; ^3^ Department of Medicine III, Ludwig Maximilian University (LMU) Munich, Munich, Germany; ^4^ Division of Medical Oncology, Netherlands Cancer Institute, Amsterdam, Netherlands; ^5^ Department of Medical Oncology, Leiden University Medical Center, Leiden, Netherlands; ^6^ Melanoma Clinic, Centre Hospitalier Universitaire Vaudois, Lausanne, Switzerland; ^7^ Department of Hematology and Oncology, University Children’s Hospital Tübingen, Tübingen, Germany

**Keywords:** tumor reactive T cells, CD137, TILs, CliniMACS Prodigy, REP, GMP compliant cell manufacturing, automation

## Abstract

Recent studies have revealed the potential of tumor-infiltrating lymphocytes (TILs) to treat solid tumors effectively and safely. However, the translation of TIL therapy for patients is still hampered by non-standardized and laborious manufacturing procedures that are expensive and produce highly variable cellular products. To address these limitations, the CliniMACS Prodigy^®^ Tumor Reactive T cell (TRT) Process has been developed. The TRT Process allows the automated isolation, transduction, and expansion of tumor-reactive T cells in a clinically compliant and closed system under GMP conditions. The TRT Process can generate tumor-reactive T cells using several methodologies which reflect clinically relevant applications. It can manage an automated Rapid Expansion Protocol (REP) using GMP-compliant reagents to generate a TIL cell product from solid tumors, including melanoma. Additionally, the TRT Process automates the closed selection of CD137-expressing TILs directly from tumor digest followed by the direct expansion of selected cells. Enriched CD137^+^ TILs could be robustly expanded even when as few as 1x10^4^ TILs were used to seed the REP phase. These data provide proof-of-concept for the isolation and expansion of tumor-reactive T cells from tumor digest in a closed, automated manner in the CliniMACS Prodigy, allowing for an efficient, simple, and reproducible manufacturing of TIL products. The direct selection of CD137^+^ TILs from tumor digest removes the need for the pre-REP phase, selects for therapeutically relevant cells, and can dramatically shorten the manufacturing time compared to conventional methods.

## Introduction

Tumor infiltrating lymphocyte (TIL) therapy has achieved durable, complete remissions in metastatic melanoma and promising signs of efficacy in solid tumors more broadly, such as lung cancer, cervical cancer and others (1–5). Despite these successes, the manufacturing processes for TIL therapies differ greatly, are largely non-automated and contain open manufacturing steps (6–9). As TIL therapy enters the age of commercialization with the FDA approval of lifileucel (Amtagvi™) in early 2024, an automated TIL manufacturing system will be required to control costs, ensure product consistency and reduce manufacturing time (10–12). Furthermore, it will be necessary to enable point-of-care and decentralized manufacturing as an option to allow the scaling of these therapies to larger numbers of patients (13).

Another significant driver of TIL variability has been the relatively low and variable amount of tumor reactivity observed in the final TIL products. Often the infused TIL product contains only a minor fraction of tumor-reactive T cells (TRTs) (14, 15). This paucity of tumor-reactive TIL in the final product necessitates higher TIL cell numbers for treatment, reduces the efficacy of the drug product and adds costs, as much of the manufacturing is contributed towards T cells which have no therapeutic effect (16). The field has sought to ameliorate this problem by selecting specific cell surface receptors on TILs, which can serve as “*in situ”* markers of tumor-specificity. PD-1 was identified as one of the first potential markers to select tumor-reactive T cells (17, 18). Additional strategies combining different surface markers to identify tumor-reactive TILs have been described including CD39^+^/CD103^+^, PD1^+^/ICOS^+^, PD1^hi^, CD39^-^/CD69^-^ and others (19–22). Comparative analysis of PD-1, CD137, CD39 and CD103 as markers of reactive TIL demonstrated that expression of CD137 (4-1BB), a T cell activation marker, was the most selective and discriminating for the enrichment of tumor reactivity. Removal of CD137 expressing cells from subsets enriched for PD1, CD39 or CD103 reduced the reactivity of the TILs within tumor digest, suggesting that CD137^+^ T cells can identify the reactive cells within these populations. Previous work has demonstrated that unlike other tumor markers, which are expressed *in situ*, CD137 can be additionally upregulated on tumor-reactive T cells after tumor digestion presumably due to TCR mediated recognition of peptide-MHC complexes on tumor cells during the co-culture (23). Thus, CD137 can serve as an antigen agnostic marker for T cell antigen engagement and therefore represents a better option for precise identification of T cells with superior tumor reactivity both *in vitro* and *in vivo* (24–28). The refinement of methodologies and procedures to better expand tumor reactive T cells is critical as the intrinsic diversity of TIL tumor recognition is one of the central rationales for its effective use, particularly in comparison to CAR- or TCR-based approaches which tend to be mono-antigenic (29, 30).

The CliniMACS Prodigy, a closed and semi-automated cell culture platform, has been used for clinical manufacturing of multiple adoptive cell therapies including CAR-T cells, T cell receptor engineered T cells, NK cells and others (31–33). The TRT Process has been developed to automatically enrich CD137^+^ tumor-reactive TILs and to expand selected cells in a rapid expansion protocol (REP). The TRT Process is GMP-compliant and can produce a cellular product from enriched material in a short 16-day process. In process control (IPC), quality control (QC) and sampling steps are included within the workflow. After expansion, the process performs a cell product wash and final formulation to obtain an infusion-ready product.

## Results

### Clinical scale rapid expansion of young TILs

**Figure 1:**
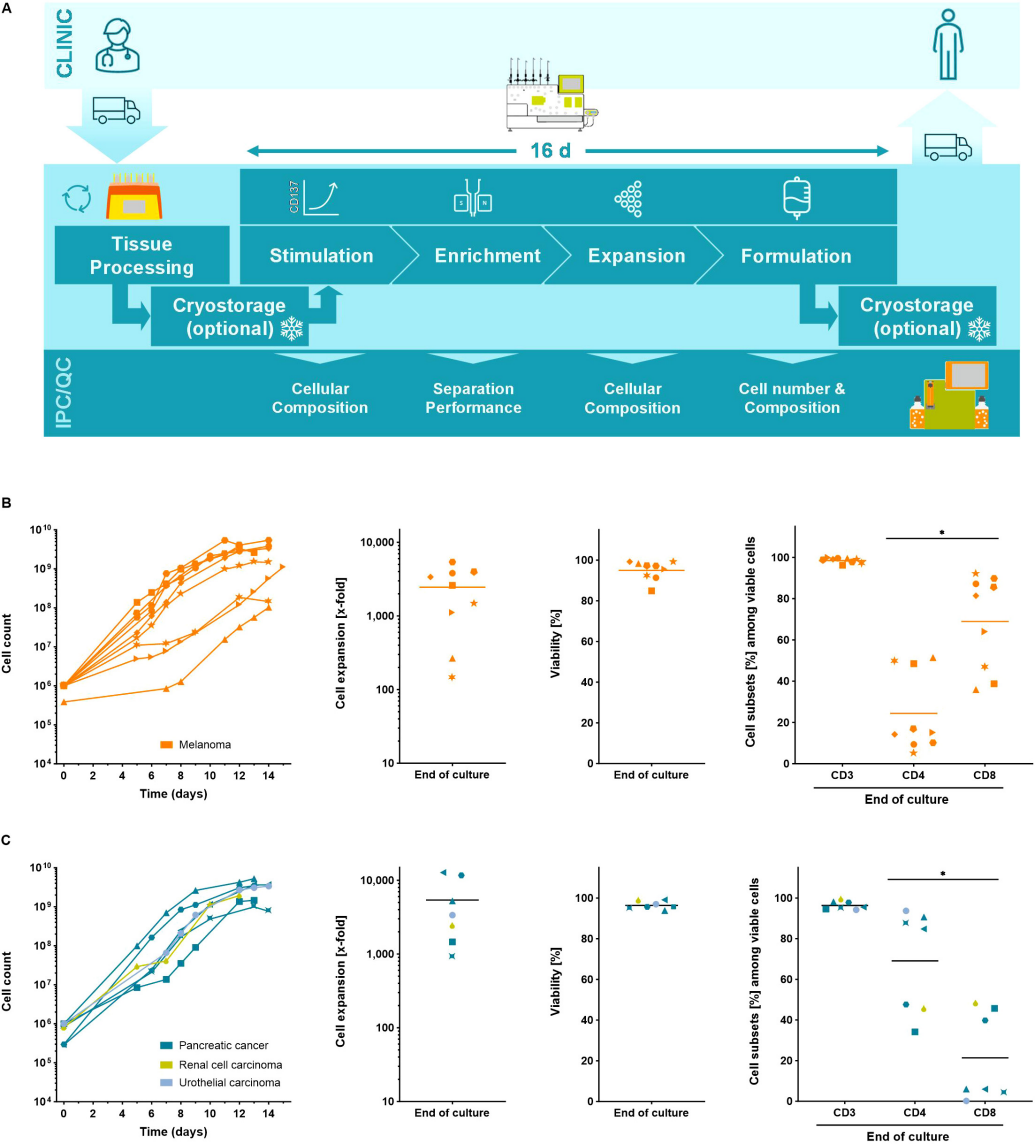
The Tumor Reactive T cell (TRT) Process using the CliniMACS Prodigy. **(A)** Schematic overview of the manufacturing process. Automated expansion of starting cell numbers of up to 2x10^6^ yTILs for 12 – 15 days. Cell counts, T cell expansion, viability and cell subsets of expanded **(B)** melanoma yTILs (n=9) and **(C)** yTILs from other solid tumor entities (n=7) were determined using flow cytometry analysis. Line marks the mean. Student's paired t-test was used to calculate significance. All p values <0.05 were considered statistically significant and are indicated as *p<0.05.

To facilitate the clinical application of TILs, the standard rapid expansion protocol was automated using the TRT Process (**Figure 1A**) (34). All handling steps and components of the REP, including addition of young TILs (yTILs) and irradiated feeder cells, cytokines, media, activation reagents, media exchanges, harvest and final product formulation were incorporated into the process. The specifications of the REP culture, such as frequency and number of feeding steps can be flexibly programmed by the user and are performed automatically by the CliniMACS Prodigy. Visual inspection of the cell culture to follow the growth of T cell clusters that form during the primary activation phase is possible using the integrated microscope camera of the device (**Supplementary Figure S1A**). The first 5 days of expansion are performed under static conditions to avoid disruption of cell-cell contacts that are critical in the early phase of the REP expansion. Shaking of the culture chamber has been implemented to provide adequate gas-exchange needed for higher cell densities during later phases of expansion. Timepoints and shaking parameters can be flexibly adjusted by the operator. IPC samples can be collected sterilely to monitor cell phenotype, cell composition, cell counts, viability, glucose and lactate levels throughout the process (**Supplementary Figures S1B, C**).

To demonstrate the utility of the TRT Process, between 2x10^5^ and 2x10^6^ yTILs derived from melanoma patients were expanded for 12-15 days in the automated REP. Using the TRT Process expansion rates of 2,453 ± 1,819-fold could be reached yielding final cell counts of 2.4x10^9^ ± 1.8x10^9^ TILs. An upper limit of ~5x10^9^ cells was found similar to other manufacturing processes of T cells on the CliniMACS Prodigy platform and is not TIL specific but rather reflects culture-density limitations and the chamber size (**Figure 1B**) (35). The final cell product was highly viable and contained predominantly CD3^+^ T cells. The ratio of CD4^+^ and CD8^+^ subsets was largely patient specific and was not affected by the expansion culture (**Supplementary Figure S1D**). yTILs from other solid tumors such as pancreatic cancer, renal cell carcinoma and urothelial carcinoma were also expanded with the TRT Process. Like yTILs from melanoma, yTILs from other solid tumors were robustly expanded to clinically relevant numbers reaching final cell counts of 0.7 – 5.3x10^9^ cells, which corresponded to expansion rates between 905- and 12,674-fold. Expanded TIL cell products from other solid tumors were viable, contained almost exclusively CD3^+^ T cells and showed patient dependent populations of CD4^+^ and CD8^+^ T cells (**Figure 1C**).

### Young TILs expanded with the CliniMACS Prodigy^®^ are reactive against autologous tumor cells *in vitro*

**Figure 2:**
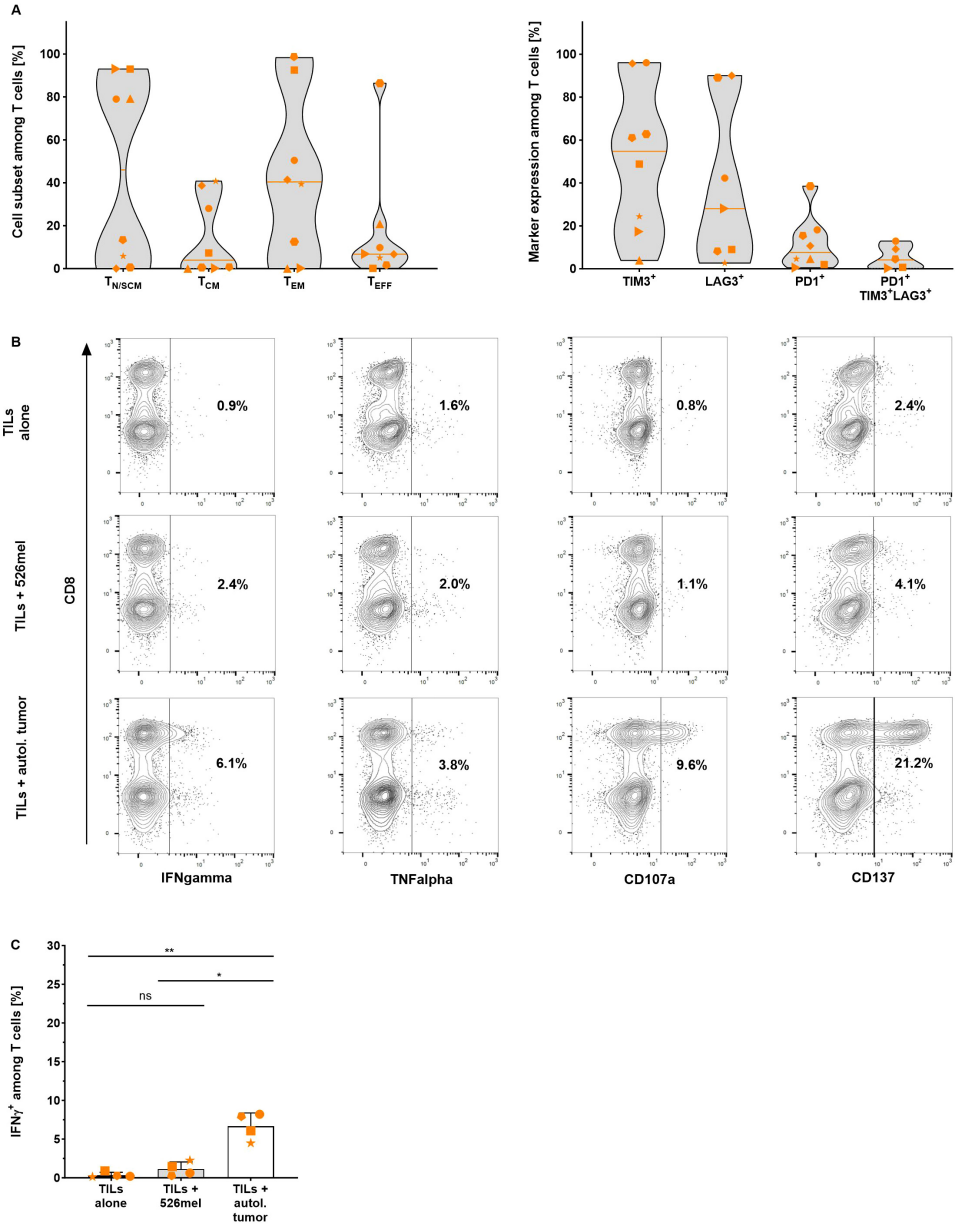
Differentiation status and specificity of TILs expanded with the TRT Process were determined using flow cytometry analysis. **(A)** Proportion of naïve or stem cell-like memory (T_N/SCM_), central memory (T_CM_), effector memory (T_EM_) and effector (T_EFF_) T cells (left graph) and expression of exhaustion markers TIM-3 (n=8), LAG-3 (n=7), PD-1 (n=8) and TIM3^+^/LAG3^+^/PD1^+^ (n=5) (right graph) among viable CD3^+^ T cells in the final product. Line marks the median (n=8). **(B)** Representative data of IFN-γ, TNF-α, CD107a and CD137 expression by TILs after an 18 hour overnight co-culture with autologous tumor cells. **(C)** Selected expanded TILs were re-stimulated with autologous or allogeneic tumor cells overnight for 18 hours and specific response was assessed by intracellular IFN-γ staining (n=4, mean with SD). Student's paired t-test was used to calculate significance. All p values <0.05 were considered statistically significant and are indicated as *p<0.05 and **p<0.01.

At the end of each expansion process, the differentiation and exhaustion status of the resulting TILs was defined using flow cytometric analysis. The final product was mainly composed of effector memory T cells (42.0 ± 38.2%, n=8) and stem cell-like memory T cells (25.5 ± 38.0%, n=8) and contained only a minor population of terminally differentiated effector T cells. The exhaustion state of the expanded TILs was defined based on expression of the co-inhibitory markers TIM3, LAG3 and PD1 (36). A considerable proportion of TILs expressed TIM-3 (51.2 ± 34.5%, n=8), followed by LAG-3 (38.4 ± 37.5%, n=7) and only very few TILs expressed PD-1 (11.8 ± 12.5%, n=8). Only low frequencies of triple-positive TIM3^+^/LAG3^+^/PD1^+^ TILs were found in the final TIL product (5.4 ± 5.5%, n=5) indicating that TILs expanded using the TRT Process are not terminally exhausted (**Figure 2A**). To prove this hypothesis the specificity and functionality of expanded cells were assessed *in vitro* by co-culturing the TILs with autologous tumor cell lines. All tested TIL products showed considerably increased IFN-γ (p=0.0038, n=5) and TNF-α (p=0.0273, n=5) production as well as upregulated expression of CD107a (p=0.0058, n=5) and CD137 (p=0.0468, n=3) upon co-culture with autologous cell lines (**Figure 2B**, **Supplementary Figure S3**). In the absence of autologous tumor cells or upon co-culture with an allogeneic melanoma cell line (526mel), little or no background activity was observed, suggesting that the TIL recognition was HLA and tumor-line specific (**Figure 2C**).

### Clinical scale stimulation, CD137 enrichment and expansion of tumor-reactive T cells

To automate the enrichment of antigen-specific T cells using the TRT Process, CD137 was selected as marker for magnetic separation based on previous findings (24, 25). As the development of the process was hampered by the scarcity of appropriate tumor samples, leukapheresis (LP) was used as a more accessible starting material. The process was mimicked using a mixture of viral peptides (PepTivator^®^ Peptide Pools) to stimulate peripheral blood cells overnight for 16 to 20 hours followed by CD137 enrichment of activated virus-specific T cells (VSTs).

**Figure 3:**
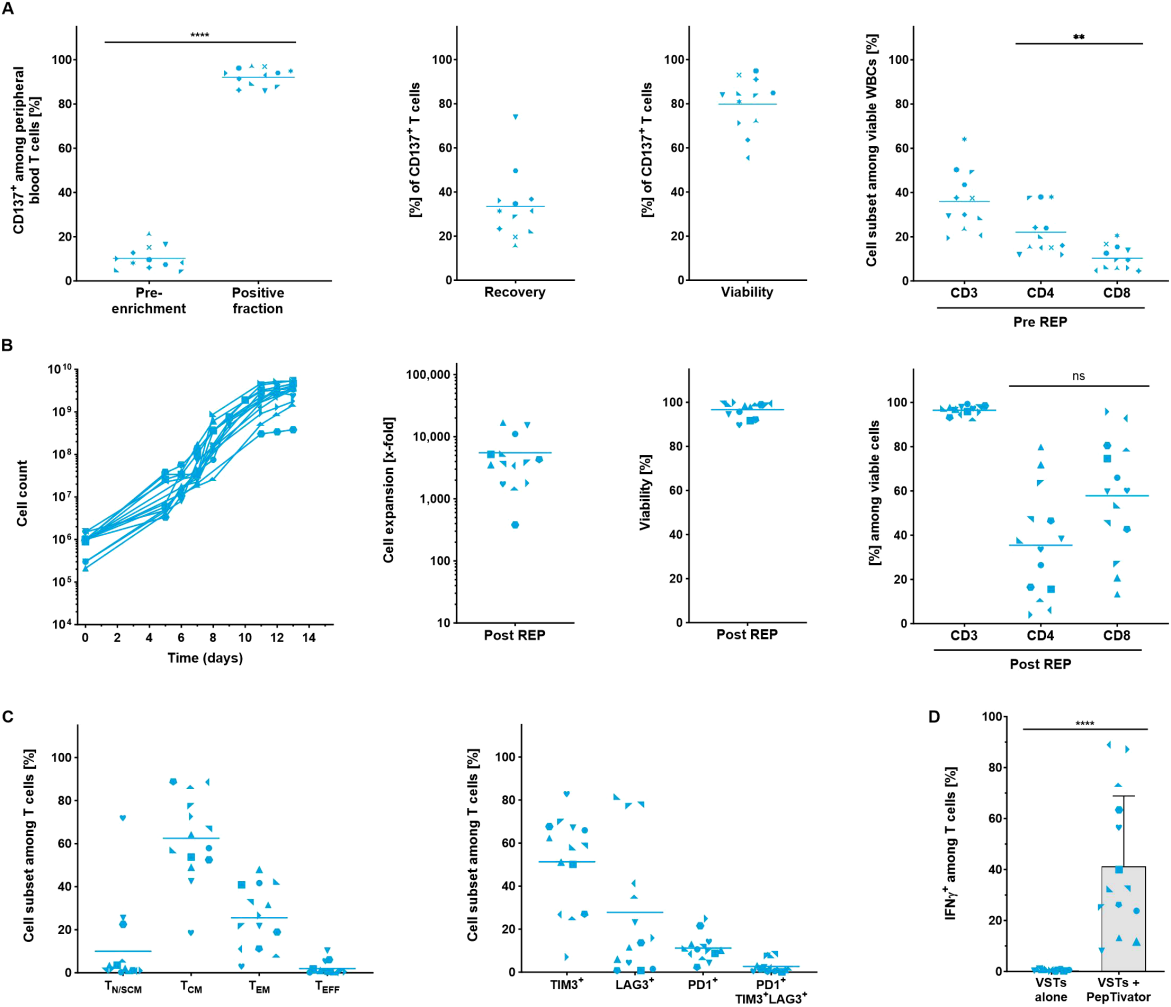
Automated stimulation, enrichment and expansion of virus-specific T cells from LP. All parameters were determined using flow cytometry analysis. **(A)** Performance of the separation. Frequency of CD137^+^ T cells was assessed in the starting material and in the positive fraction after enrichment. Recovery of CD137^+^ T cells, viability and cellular composition in the positive fraction was evaluated after enrichment (n=12). **(B)** Automated expansion of VSTs. T cell expansion kinetics, viability and the cellular composition of expanded VSTs (n=14). **(C)** Differentiation and exhaustion status among viable CD3^+^ T cells at the end of the expansion culture (n=14). For A-C, line marks the mean. **(D)** IFN-γ production of expanded VSTs and after re-stimulation with virus-specific PepTivator Peptide Pools (n=14, mean with SD). Student's paired t-test was used to calculate significance. All p values <0.05 were considered statistically significant and are indicated as **p<0.01 and ****p<0.0001.

Despite low starting frequencies, a high purity of CD137^+^ T cells (92.1 ± 4.0%) could be reached following the magnetic isolation. On average, the recovery of CD137^+^ T cells within the positive fraction was 33.4 ± 15.7% while the viability was 79.8 ± 12.1% with some donor dependent variability (**Figure 3A**). The enriched CD137^+^ fraction contained 36.0 ± 13.6% T cells, with a slightly higher proportion of CD4^+^ T cells. The remaining contaminating populations in the enriched fraction were CD137^+^ neutrophils, eosinophils and monocytes (**Supplementary Figure S3**).

Low input numbers of CD137-enriched T cells (between 2x10^5^ – 1.5x10^6^ T cells) were expanded for 13 – 14 days to final cell counts of 1.4 – 5.4x10^9^ cells, corresponding to expansion rates of 1,105- to 16,864-fold. The viability of the cells at the end of the culture was consistently high (**Figure 3B**). Notably, despite the heterogenous starting cell population, the expanded cells contained > 90% CD3^+^ T cells. On average, the frequencies of CD4^+^ and CD8^+^ T cells were well balanced with slight bias towards the CD8^+^ T cells (35.5 ± 24.3% CD4^+^ and 57.8 ± 25.9% CD8^+^). Most T cells in the final product showed central memory (62.5 ± 19.5%) and effector memory (25.6 ± 14.4%) phenotype. A considerable proportion of expanded VSTs expressed TIM-3 (51.3 ± 21.8%) but only moderate populations stained positive for LAG-3 (27.8 ± 30.1%) and PD-1 (11.2 ± 6.1%). Like expanded yTILs, the expression of the exhaustion markers seemed not to be co-regulated and consequently only low frequencies of triple positive VSTs (2.7 ± 2.9%) could be detected. (**Figure 3C**).

To confirm the specificity and function of VSTs generated by the TRT Process the production of IFN-γ among CD3^+^ T cells was determined in the final product (**Figure 3D**). To this end, expanded VSTs were re-stimulated with the same virus-specific PepTivators which were initially used for stimulation and enrichment (**Figure 3D**). All VST preparations significantly upregulated IFN-γ in the presence of the viral PepTivators (VSTs p<0.0001, n=14) whereas no background production could be detected in non-stimulated samples indicating that the TRT Process with its CD137 enrichment step enables the isolation and expansion of antigen-specific T cells.

**Figure 4:**
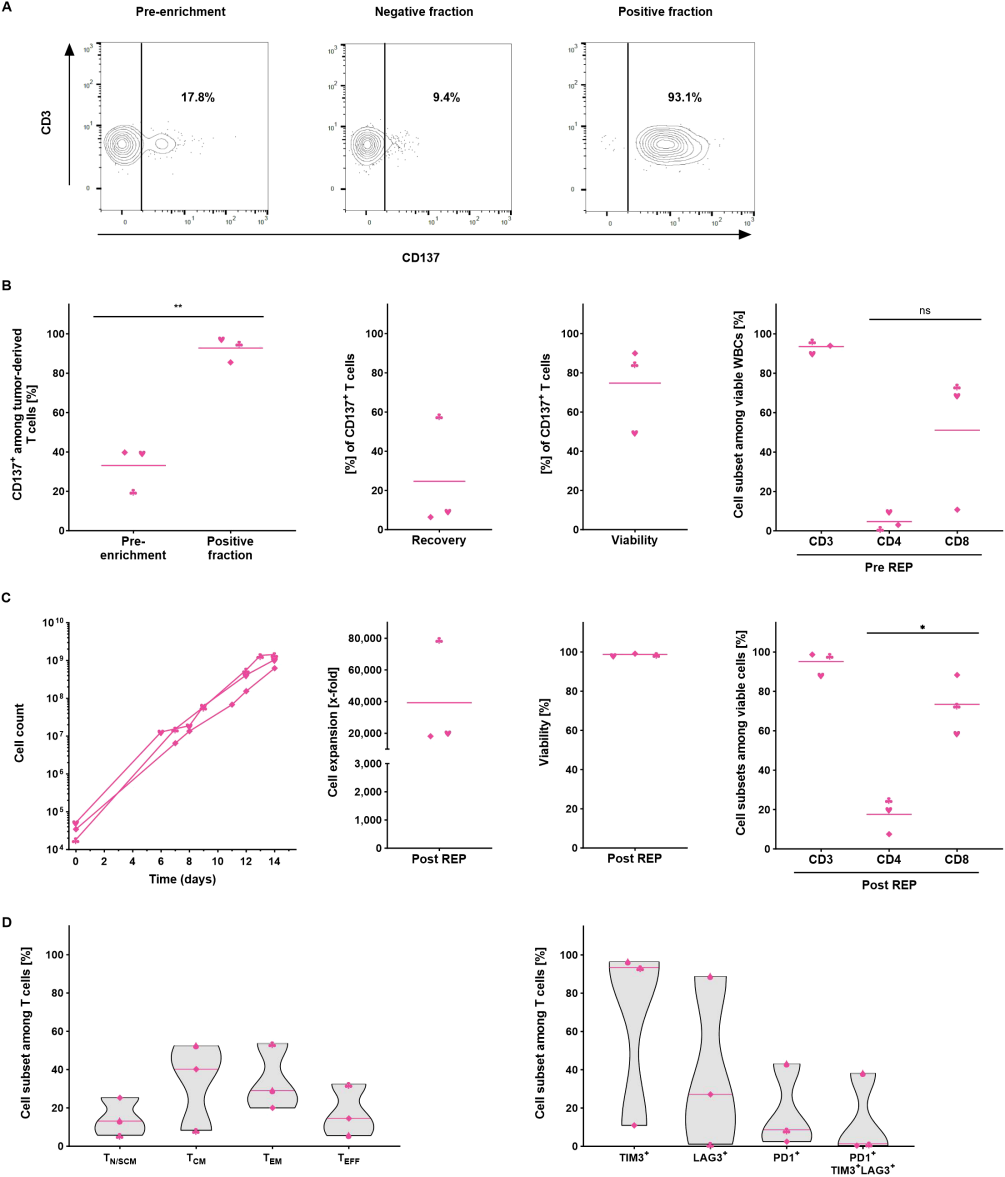
Automated manufacturing of tumor-reactive T cells from melanoma samples (n=3). All parameters were determined using flow cytometry analysis. **(A)** Representative flow cytometry analysis of the CD137 expression in the starting material, in the negative and in the positive fraction after enrichment. **(B)** Performance of the separation. Frequency of CD137^+^ T cells was assessed in the starting and in the positive fraction after enrichment. Recovery of CD137^+^ T cells, viability and cellular composition in the positive fraction after enrichment. **(C)** Automated expansion of TRTs. T cell expansion, viability and the cellular composition of expanded TRTs at the end of the culture. For B+C, line marks the mean. **(D)** Differentiation and exhaustion status among viable CD3^+^ T cells at the end of the expansion culture. Line marks the median. Student's paired t-test was used to calculate significance. All p values <0.05 were considered statistically significant and are indicated as *p<0.05 and **p<0.01.

After confirming the performance of the TRT Process to enrich and expand VSTs from peripheral blood, the process was validated using melanoma tumor samples. Enzymatically digested tumor tissue containing both tumor and T cells was cultured overnight for 16 to 18 hours and subjected to the CD137 magnetic cell separation. After enrichment, the frequency of CD137^+^ cells among viable T cells was evaluated to determine the enrichment performance (**Supplementary Figure S4**, **Figure 4A**). The CD137 isolation of TRTs yielded consistently high purity with CD137 frequencies among T cells increasing from 33.1 ± 11.3% prior to enrichment to 92.8 ± 6.4% following enrichment. The recovery of CD137^+^ T cells within the positive fraction was 24.6 ± 29% and the viability of the TRTs was 74.7 ± 22.0%. The enriched CD137^+^ cell fraction consisted of 93.5 ± 3.2% T cells on average, with higher proportion of CD8^+^ T cells (**Figure 4B**).

Due to the small size of the tumor samples, the overall number of enriched CD137^+^ TRTs was rather low and consequently only 1.8 – 5.7x10^4^ TRTs were subjected to the rapid expansion. Despite the exceptionally low starting cell numbers, cells could be expanded with consistently high viability to 6.3x10^8^ – 1.5x10^9^ T cells which corresponded to expansion rates of 18,158- to 79,325-fold. Expanded TRTs had high T cell purities of > 90% CD3^+^ T cells for two of the runs while the third run showed a slightly lower frequency of 88.3% (**Figure 4C**). On average, higher percentages of CD8^+^ T cells were seen at the end of the expansion (17.6 ± 9.1% CD4^+^ and 73.4 ± 24.8% CD8^+^). The predominant subpopulations among expanded TRTs displayed a central memory (33.6 ± 22.8%) and effector memory (34.2 ± 17.4%) phenotype. Like expanded yTILs, expanded TRTs were largely TIM-3^+^ (66.9 ± 48.5%) but expressed less LAG-3 (39.0 ± 35.0%) and PD-1 (18.0 ± 21.9%) and only a minority co-expressed all three markers (13.3 ± 21.5%) (**Figure 4D**).

### TRTs manufactured using the CliniMACS Prodigy^®^ show superior anti-tumor reactivity *in vitro*

**Figure 5:**
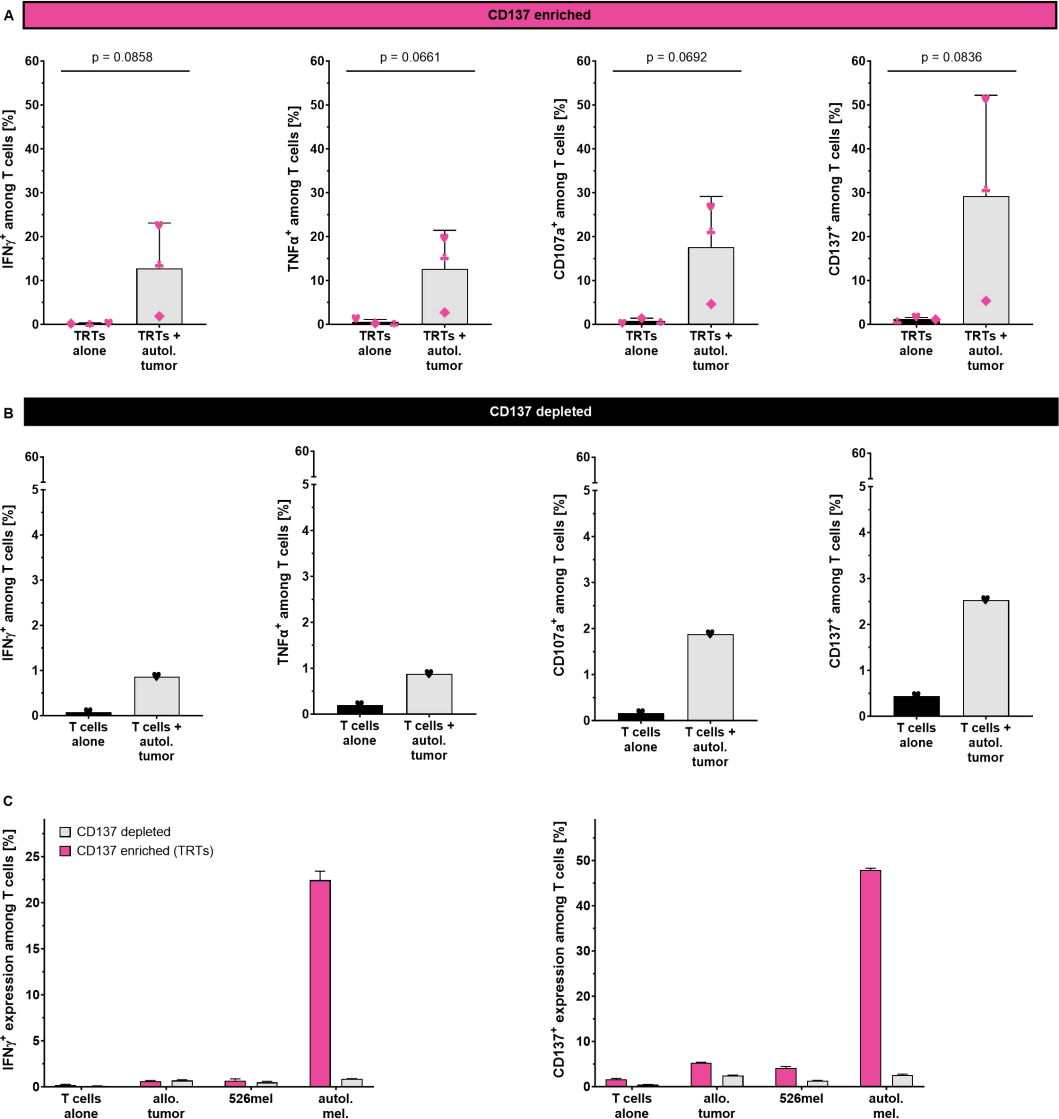
Tumor specificity of CD137-enriched and expanded TRTs as determined by flow cytometry analysis. **(A)** Reactivity of CD137-enriched TRTs (n=3, mean with SD) and **(B)** CD137-depleted TILs against the autologous tumor cell line. **(C)** Reactivity of CD137-enriched TRTs and CD137-depleted TILs from one selected donor against autologous and allogeneic tumor cells (technical triplicates, mean with SD). Student's paired t-test was used to calculate significance. All p values <0.05 were considered statistically significant.

To confirm the tumor-specific reactivity of the CD137-enriched TRTs generated using the TRT Process, autologous tumor cell lines were generated and used as target cells. For direct comparison, matched CD137-depleted TILs were expanded in parallel using the same automated REP process (**Supplementary Figure S5**). CD137^+^ TRTs and CD137^-^ TILs were co-cultured with autologous tumor cell lines overnight for 18 hours and subsequently analyzed for cytokine production, expression of the degranulation marker CD107a and the activation marker CD137. As additional negative control, TRTs were cultured in the absence of target tumor cells. CD137^+^ TRTs from all three samples showed considerably increased production of IFN-γ (p=0.0858, n=3) and TNF-α (p=0.0661, n=3) as well as upregulation of CD107a (p=0.0692, n=3) and CD137 (p=0.0836, n=3) upon co-culture with autologous tumor cell lines (**Figure 5A**). In contrast, CD137^-^ TILs did not show any activation after co-culture with autologous tumor cell lines suggesting that tumor reactivity was almost exclusively attributed to the CD137^+^ fraction (**Figure 5B**).

To further confirm tumor specificity of the generated TRTs, one selected TRT preparation was tested against allogeneic patient derived tumor cell line as well as the HLA-A2^+^ melanoma cell line 526mel (**Figure 5C**). A considerable proportion of CD137-enriched TRTs secreted IFN-γ in response to autologous tumor cells, whereas no response could be seen against allogeneic tumor cells and the 526mel cell line. The corresponding expanded CD137^-^ fraction showed only negligible responses against both autologous and allogeneic tumor cells with no preference towards the autologous tumor cells. Similar trends with even higher frequencies were observed for the upregulation of CD137.

## Discussion

As TIL therapy enters commercialization, streamlining manufacturing processes is crucial to ensure patient access and reduce costs. Current TIL manufacturing processes are labor-intensive, non-standardized, and often involve many open or non-automated steps. Additionally, only a small fraction of the produced TILs mediate therapeutic activity, wasting resources on the growth of irrelevant bystander cells. These factors contribute to a non-standardized, expensive, and variable cell product and the need of extraordinary cell numbers for therapeutical treatment. Therefore, incorporating an enrichment step for tumor-reactive T cells and transitioning TIL manufacturing to an automated and closed platform could help reduce costs and ensure a consistent, tumor-specific product, thereby expanding TIL therapy access to a broader cohort of patients.

The CliniMACS Prodigy is a GMP-compliant, “all-in-one” cell culture platform that has been successfully used to manufacture T cells, NK cells, and other cell types for therapeutic applications (31–33, 37). The TRT Process supports the modular and flexible manufacturing of TILs. It includes dedicated software, a closed single-use tubing set, and GMP-grade reagents for the GMP-compliant manufacturing of bulk or CD137^+^ TILs in as few as 16 days. The TRT Process is fully customizable: the user can adapt the timings and frequency of all steps, and modify culture conditions as desired, by using e.g. alternative media compositions, cytokine cocktails, or small molecules to optimize the attributes of the desired final product or to alter the expansion time.

The automated rapid expansion of TILs using the TRT Process was able to robustly expand yTILs from melanoma and other solid tumor indications. At the end of the 14-day culture period, TILs were highly viable, had a T_CM/EM_ skewed phenotype, and exhibited robust fold expansion. In general, the TILs produced by the TRT Process are similar to those reported by other groups using standard processes (8–10), suggesting that the automated and closed methodology successfully replicates the traditional REP process. However, the TRT Process has a limited maximum cell number of approximately 5x10^9^ TILs. Since many current TIL studies using expanded bulk TILs dose 1x10^10^ or even up to 1x10^11^ TILs for treatment, future work will seek to increase the final yield of the TRT Process by adapting the process to the newly developed large tubing set, which contains a larger cultivation chamber and has already demonstrated an increase in CAR T cell yield from 5x10^9^ to approximately 1 – 2x10^10^ (35). A similar increase in TIL yield with the large-scale tubing set should suffice for most future clinical TIL applications as the requirements for TIL cell numbers for clinical application are expected to decline. Ongoing research in academia and industry to improve TIL manufacturing processes, enhance TIL tumor-reactivity, and endow TILs with genetic modifications to avoid immune suppression will likely increase TIL efficacy (38–43).

The TRT Process offers a GMP-compliant methodology for the automated enrichment of CD137^+^ TRTs directly from enzymatically digested tumor samples. Although several TIL selection strategies are compatible with FACS-based sorting (20–23), CD137 as a single marker was amenable to automated magnetic selection in the CliniMACS Prodigy. This was first demonstrated with virus-reactive cells from peripheral blood and then with TILs from tumor digest. The selection was robust and could facilitate the expansion of antigen-specific T cells with virus or tumor reactivity without the need for a pre-REP. A similar process was already described earlier for clinical scale manufacturing of tumor/neoantigen-specific TILs (44). In this workflow, autologous tumor cell lines were used for re-stimulation of outgrown yTILs. Despite the pioneering character of this work, the laborious procedure and the fact that generation of autologous tumor cell lines is highly unreproducible prevented timely and consistent clinical scale manufacturing of TRTs. The TRT Process overcomes these challenges by isolating TRTs directly from tumor digest after a short pre-culture phase. The results support previous reports underscoring the benefit of CD137 as an enrichment marker (24–28). Furthermore, it was demonstrated that depletion of CD137^+^ cells from the tumor digest results in a loss of anti-tumor activity in the final TIL product, which was also consistent with previous findings (23). Thus, CD137 enrichment might not only select for tumor-specific but also functional, tumor-reactive TILs (23, 25, 45). This could improve and standardize the therapeutic activity of the cellular product while reducing the number of cells required for efficacious treatment.

A secondary advantage of CD137 enrichment directly from tumor digest is that it facilitates omitting the pre-REP culture and therefore enables the manufacturing of a clinically relevant TIL product within only 16 days, making it one of the shortest processes for TIL manufacturing currently described. It has been reported that tumor-mediated factors can negatively influence the generation of TIL within tumor fragments during the pre-REP culture (46) and that tumor-specific clonotypes can be lost by overgrowth of TRTs by irrelevant bystander T cells with greater fitness (29, 47). Thus, bypassing the pre-REP may serve to release the TRTs sooner from the suppressive microenvironment, facilitating a more reactive and fit cellular product. The viability of TRTs produced by this process is similar to other reports of clinical TIL products but exploration of *ex* vivo interventions, such as use of optimized media, alternative combinations of cytokines and small molecules during T cell expansion, could yield fitter, more viable cell products (48–50).

Future efforts will focus on adding more modules to the TRT Process to automate and standardize various manufacturing schemes and protocols currently used in academic and industrial settings. This includes integrating FACS-based cell sorting or connectivity to modules that facilitate alternative TIL culture methodologies, such as outgrowth of TILs from tumor fragments or tumor digest. Parallel efforts aim to optimize lentiviral transduction and non-viral genetic engineering approaches as genetic modification of TILs continues to gain interest. Importantly, *in vivo* description of the CD137^+^ TRT Process is the subject of ongoing research using autologous, orthotopic PDXv2.0 mouse models and will be reported in a future publication (51). Integrating these varied TIL manufacturing processes into an automated and standardized workflow will require time, experimentation and collaboration with the larger preclinical and translational TIL research communities.

A clinical translation of the TRT Process without CD137 enrichment is currently ongoing with one study (NCT04812470) manufacturing yTILs for local administration in uveal melanoma liver metastases. Additional clinical trials employing the full TRT Process and enriched CD137^+^ TRTs are projected to begin in early 2026.

## Methods

### Preparation of starting cell sources for stimulation, expansion and functional analysis

To prepare feeder cells from frozen LP or PBMCs, cells were thawed, washed with an excess of TexMACS™ GMP Medium supplemented with 3% (v/v) heat-inactivated AB serum (Cat. no. H4522-100ML, Sigma-Aldrich) and subsequently irradiated (40 Gy). To obtain tumor digest, tumor samples were cut into small pieces before processing them using the Tumor Dissociation Kit in combination with the gentleMACS™ Dissociator according to manufacturer instructions. Young tumor-infiltrating lymphocytes were generated by either culturing of a tumor fragment or tumor digest in TexMACS GMP Medium supplemented with 3-5% AB serum (Cat. no. H4522-100ML, Sigma-Aldrich), antibiotics and 6,000 IU/mL MACS^®^ GMP Recombinant Human IL-2. Fresh tumor digest and yTILs were cryopreserved in heat-inactivated AB serum supplemented with 10% DMSO. Tumor cells were cultured in RPMI 1640 (Cat. no. L0501-500, Biowest) supplemented with 10% FBS (Cat. no. BS-2022-500, Catus Biotech GmbH), 2 mM L-Glutamine (Cat. no. BE17-605E, Lonza) and antibiotics. If not specified otherwise, reagents and material were obtained from Miltenyi Biotec.

### Automated stimulation, enrichment and expansion using the TRT Process

T cell stimulation, CD137 enrichment and expansion of cells were performed using the CliniMACS Prodigy^®^ Tubing Set 520 and the TRT Process. CliniMACS^®^ PBS/EDTA Buffer containing 0.5% (v/v) human serum albumin (HSA) and TexMACS GMP Medium supplemented with 3% (v/v) heat-inactivated AB serum (Cat. no. H4522-100ML, Sigma-Aldrich) were prepared and starting cells were transferred into a 150 mL bag. The tubing set was installed according to the instructions and buffer, media and up to 1x10^9^ viable WBCs (LP) or viable cells (tumor material) were attached to the tubing set by sterile welding. Leukapheresis from healthy donors was stimulated with multiple virus-specific PepTivator Peptide Pools (MACS^®^ GMP PepTivator^®^ AdV5 Hexon, MACS^®^ GMP PepTivator^®^ HCMV pp65, MACS^®^ GMP PepTivator^®^ EBV Select, MACS^®^ GMP PepTivator^®^ HPV16-E6 and MACS^®^ GMP PepTivator^®^ HPV16-E7) at the recommended concentration of 0.6 nmol/mL for each PepTivator Peptide Pool while tumor material was stimulated by co-culture of T cells and tumor cells (contained in the tumor digest). A sample was taken after the overnight stimulation culture (between 16 to 20 hours) to assess the CD137 frequency. CliniMACS^®^ CD137 GMP Biotin and CliniMACS^®^ Anti-Biotin GMP MicroBeads were used for magnetic labelling as recommended in the respective data sheets. Sampling was performed after labelling and after enrichment to evaluate frequencies of CD137^+^ cells. For expansion, TexMACS GMP medium supplemented with 3,000 IU/mL MACS GMP Recombinant Human IL-2 and 3% (v/v) heat-inactivated AB serum (Cat. no. H4522-100ML, Sigma-Aldrich) was prepared and attached to the tubing set to replace the medium after the stimulation culture. Between 0.01 – 2x10^6^ CD3^+^ responder cells (TRTs or TILs) were cultured with 4x10^8^ irradiated feeder cells and T cell activation reagent (MACS^®^ GMP CD3 pure). After the first medium bag was empty (usually before day 8), the concentration of AB serum was diluted by using TexMACS GMP Medium containing IL-2 but lacking AB serum for further media exchanges. Sterile sampling was performed in regular intervals starting on day five to assess the cell count, viability, glucose and lactate values. For the harvest, cells were automatically rebuffered and transferred into a target cell bag. If not specified otherwise, reagents and material were obtained from Miltenyi Biotec.

### In-process controls

Samples for flow cytometric analysis were taken from unmanipulated tumor material, after stimulation, after CD137 enrichment and at different days during expansion culture. Samples were acquired by flow cytometry using the MACSQuant^®^ Analyzer 10 and different parameters were analyzed using the MACSQuantify™ Software. The cellular composition was assessed before and after CD137 separation and the separation performance was determined. During the initial static activation phase, the T cell activation can be monitored using the integrated camera of the CliniMACS Prodigy. During expansion, total cell count and viability were analyzed by flow cytometry while the glucose and lactate concentration of the medium were determined with the Accutrend^®^ Plus System (Cat. no. PZN 1696541, Roche) using the BM-Lactate (REF 03012654, measurement range 0.8−22 mmol/L) and Accutrend Glucose (REF 11447475, measurement range 20-200 mg/dL) test strips. For cell counts and cellular composition during stimulation and enrichment, the 8-Color Immunophenotyping Kit (CD19-PE-Vio^®^ 770 was exchanged for CD20-PE-Vio^®^ 770) was used, according to the manufacturer’s instructions (**Supplementary Figure S6A**). For the CD137 staining, cells were stained in CliniMACS PBS/EDTA Buffer + 0.5% HSA according to the manufacturer’s instructions using the following fluorochrome-conjugated monoclonal antibodies and reagents: CD3-FITC, CD4-APC, CD8-VioBlue^®^, CD45-VioGreen™, CD137-PE or Labeling Check Reagent-PE and 7-AAD Staining Solution. CD137 was stained with a CD137 fluorochrome-conjugated antibody before and with Labeling Check Reagent after magnetic labelling. The purity was evaluated among viable CD4/CD8^+^ T cells and the recovery was calculated as follows:


$$\begin{array}{c} corrected\;recovery[\%]=\\ \;\frac{{\left(total\;viable\;CD4/CD8^{+}CD137^{+}cells\right)}_{pos}}{{\left(total\;viable\;CD4/CD8^{+}CD137^{+}cells\right)}_{pos}+{\left(total\;viable\;CD4/CD8^{+}CD137^{+}cells\right)}_{neg}} \end{array}$$

For measurement of cell counts and viability during the expansion, Propidium Iodide was added to the cells directly before acquisition (**Supplementary Figure S6B**). For the phenotype staining, cells were stained in CliniMACS PBS/EDTA Buffer + 0.5% HSA according to the manufacturer’s instructions using the following fluorochrome-conjugated monoclonal antibodies: CD3-FITC, CD4-VioBlue^®^, CD8-VioGreen™, CD27-VioBlue^®^, CD28-APC, CD45RA-APC-Vio^®^770, CD62L-PE, CD95-PE-Vio^®^770, CD223 (LAG3)-PE, CD279 (PD1)-PE-Vio^®^770 and CD366 (TIM3)-APC. Propidium iodide was added directly before acquisition to exclude dead cells. To characterize T cell differentiation, cells were classified into naïve (CD45RA^+^, CD62L^+^, CD95^-^), stem cell-like memory (CD45RA^+^, CD62L^+^, CD95^+^), central memory (CD45RA^-^, CD62L^+^), effector memory (CD45RA^-^, CD62L^-^) and effector (CD45RA^+^, CD62L^-^) cells. If not specified otherwise, antibodies, reagents and material were obtained from Miltenyi Biotec.

### Intracellular cytokine staining and CD107a mobilization shift assay

After rapid expansion, the cell functionality was analyzed by flow cytometry based on the procedure described in previous publications (52, 53). Briefly, 1 – 2x10^5^ expanded TILs or TRTs cells were stimulated with 1 – 2x10^5^ cells from the corresponding autologous or allogeneic tumor cell line. CD107a-VioBlue^®^ was added at the beginning of the stimulation period and BD GolgiPlug™ (contains brefeldin A; Cat. no. 555029, BD Biosciences) and BD GolgiStop™ (contains monensin; Cat. no. 554724, BD Biosciences) were added after 1 hour at the recommended concentration. After 18 hours, cells were stained in CliniMACS PBS/EDTA Buffer + 0.5% HSA according to the manufacturer’s instructions using the following fluorochrome-conjugated monoclonal antibodies: CD4-VioGreen™ and CD8-FITC. For the staining of intracellular markers, the Inside Stain Kit was used according to the manufacturer’s instructions. Cells were fixed and permeabilized and the following fluorochrome-conjugated monoclonal antibodies were used: CD137-PE, CD154-APC, IFN-gamma-APC-Vio^®^770 and TNF-alpha-PE-Vio^®^770. The production of IFN-gamma and TNF-alpha as well as the expression of CD107a, CD137 and CD154 were assessed among CD4^+^ and/or CD8^+^ cells. If not specified otherwise, antibodies, reagents and material were obtained from Miltenyi Biotec.

### Statistical methods

Statistical analysis was performed using GraphPad Prism software. Differences between groups and the corresponding p values were determined by parametric, Student's paired t-tests (one-tailed or two-tailed, as appropriate). All p values <0.05 were considered statistically significant and are indicated as *p<0.05, **p<0.01, ***p<0.001 and ****p<0.0001.

## Data Availability

The datasets presented in this article are not readily available because data presented in this publication is part of a commercial product development at Miltenyi Biotec and thus might include company’s knowhow that cannot be disclosed to the public. Requests to access the datasets should be directed to Andreasd@miltenyibiotec.de.

## Supplementary material

Supplementary Figure 1 Parameters monitored during the automated expansion of young TILs from melanoma patients using the TRT Process. **(A)** Microscope pictures can be taken as an indirect measurement of the activation efficiency using the integrated microscope camera of the CliniMACS Prodigy (Scale bars = 100 μm). Pictures of yTILs during the expansion culture were taken on day 0 (upper picture) and 5 (lower picture). **(B)** Lactate (dotted line indicates upper detection limit) and **(C)** glucose levels were monitored during the expansion culture using the Accutrend^®^ Plus System to guide media exchanges. **(D)** Ratio of CD4^+^ and CD8^+^ T cells among CD3^+^ TILs pre and post REP as determined by flow cytometry analysis (n=5, bars represent the mean). Student’s paired t-test was used to calculate significance. All p values <0.05 were considered statistically significant.

Supplementary Figure 2 Functionality of TILs expanded with the TRT Process was assessed via flow cytometry analysis using IFN-γ (n=5) and TNF-α (n=5) production as well as CD107a (n=5) and CD137 (n=3) expression after an 18 hour overnight co-culture with autologous tumor cells (mean with SD). Student's paired t-test was used to calculate significance. All p values <0.05 were considered statistically significant and are indicated as *p<0.05 and **p<0.01. Donors ★, n, l and Á are also shown in **Figure 2C**.

Supplementary Figure 3 Characterization of CD137 enriched virus-specific T cells from LP. The cellular composition in the positive fraction was evaluated after enrichment using flow cytometry analysis (n=12). Line marks the mean.

Supplementary Figure 4 Representative flow cytometry analysis of the CD137 expression of a tumor digest sample in the starting material (pre-enrichment), negative and positive fraction after enrichment. **(A)** Gating strategy for the analysis of the CD137 enrichment performance: Dead and apoptotic cells are excluded by a gate around viable 7-AAD negative cells. Cells are separated from debris via forward scatter (FSC) and side scatter (SSC). Lymphocytes are defined as CD45^+^ SSC^low^ cells and further gated for CD3^+^ to identify all T cells. The frequency of CD137^+^ cells is determined within gated viable CD3^+^ T cells. **(B)** Example of CD137-enrichment performance. CD137 frequency pre-enrichment, in the positive and in the negative fraction of the same donor.

Supplementary Figure 5 Automated expansion of the positive (TRTs) and negative fraction from the CD137 enrichment of the same donor. T cell expansion, viability and the cellular composition of expanded cells was evaluated at the end of the culture using flow cytometry analysis.

Supplementary Figure 6 Representative gating strategy for the determination of cell counts, viability and cellular composition using flow cytometry analysis. **(A)** The 8-Color Immunophenotyping Kit is used for viable T cell counts, T cell viability and cellular composition during stimulation and enrichment (exemplary flow data of an LP sample used for enrichment of virus-specific T cells): For elimination of doublets, a gate is set around single cells in forward scatter area (FSC-A) versus forward scatter height (FSC-H). To identify the major circulating blood cell types, CD45 is used to target all leukocytes. These cells are further separated from debris via forward scatter (FSC) and side scatter (SSC). Dead and apoptotic cells are excluded by a gate around viable 7-AAD^-^ cells. B cells are defined as CD20^+^ while CD3 is used to identify T cells. The T cells are further divided into CD4^+^ and CD8^+^ T cells. Monocytes are identified based on their CD14 expression. Among the non-monocyte population, cells are separated into CD16^+^/SSC^high^ neutrophils, CD16^–^/SSC^high^ eosinophils as well as a CD16^-/dim^/SSC^low^ population. The CD16^-/dim^/SSC^low^ population is subdivided based on CD3 and CD56 to distinguish the CD56^+^ NK cells and a CD3^+^CD56^+^ NK T cell population. **(B)** Samples stained with Propidium Iodide are used for viable cell counts and viability during the expansion (exemplary flow data of expanded yTILs on day 14): Cells are separated from debris via forward scatter (FSC) and side scatter (SSC). Dead and apoptotic cells are excluded by a gate around viable PI^-^ cells.
