# Supplementary figures and images for: Clinical-scale, modular manufacturing of tumor-reactive TILs using a closed and automated culture system
Source: Front Immunol. 2024 Dec 9;15:1483254. doi: 10.3389/fimmu.2024.1483254 (PMC11664263; doi:10.3389/fimmu.2024.1483254)

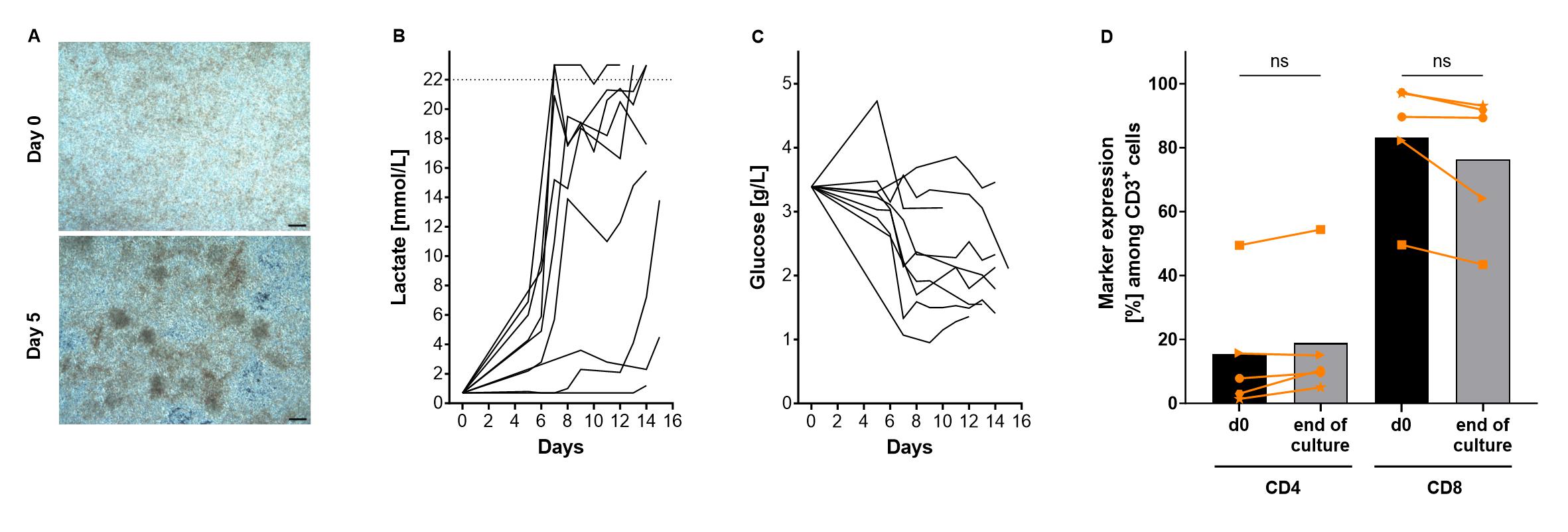

Supplement: Supplementary Figure 1 — Parameters monitored during the automated expansion of young TILs from melanoma patients using the TRT Process. (A) Microscope pictures can be taken as an indirect measurement of the activation efficiency using the integrated microscope camera of the CliniMACS Prodigy (Scale bars = 100 μm). Pictures of yTILs during the expansion culture were taken on day 0 (upper picture) and 5 (lower picture). (B) Lactate (dotted line indicates upper detection limit) and (C) glucose levels were monitored during the expansion culture using the Accutrend® Plus System to guide media exchanges. (D) Ratio of CD4+ and CD8+ T cells among CD3+ TILs pre and post REP as determined by flow cytometry analysis (n=5, bars represent the mean). Student’s paired t-test was used to calculate significance. All p values <0.05 were considered statistically significant. [file Image1.jpg]

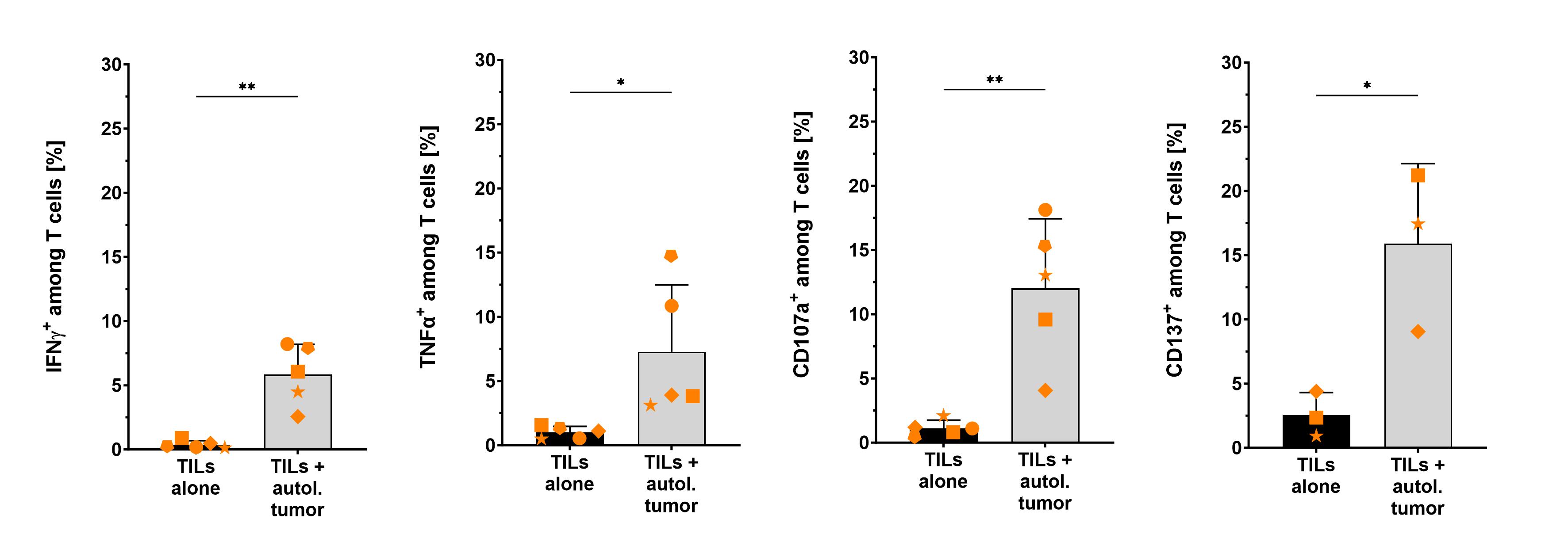

Supplement: Supplementary Figure 2 — Functionality of TILs expanded with the TRT Process was assessed via flow cytometry analysis using IFN-γ (n=5) and TNF-α (n=5) production as well as CD107a (n=5) and CD137 (n=3) expression after an 18 hour overnight co-culture with autologous tumor cells (mean with SD). Student's paired t-test was used to calculate significance. All p values <0.05 were considered statistically significant and are indicated as *p<0.05 and **p<0.01. Donors ★, n, l and Á are also shown in Figure 2C . [file Image2.jpeg]

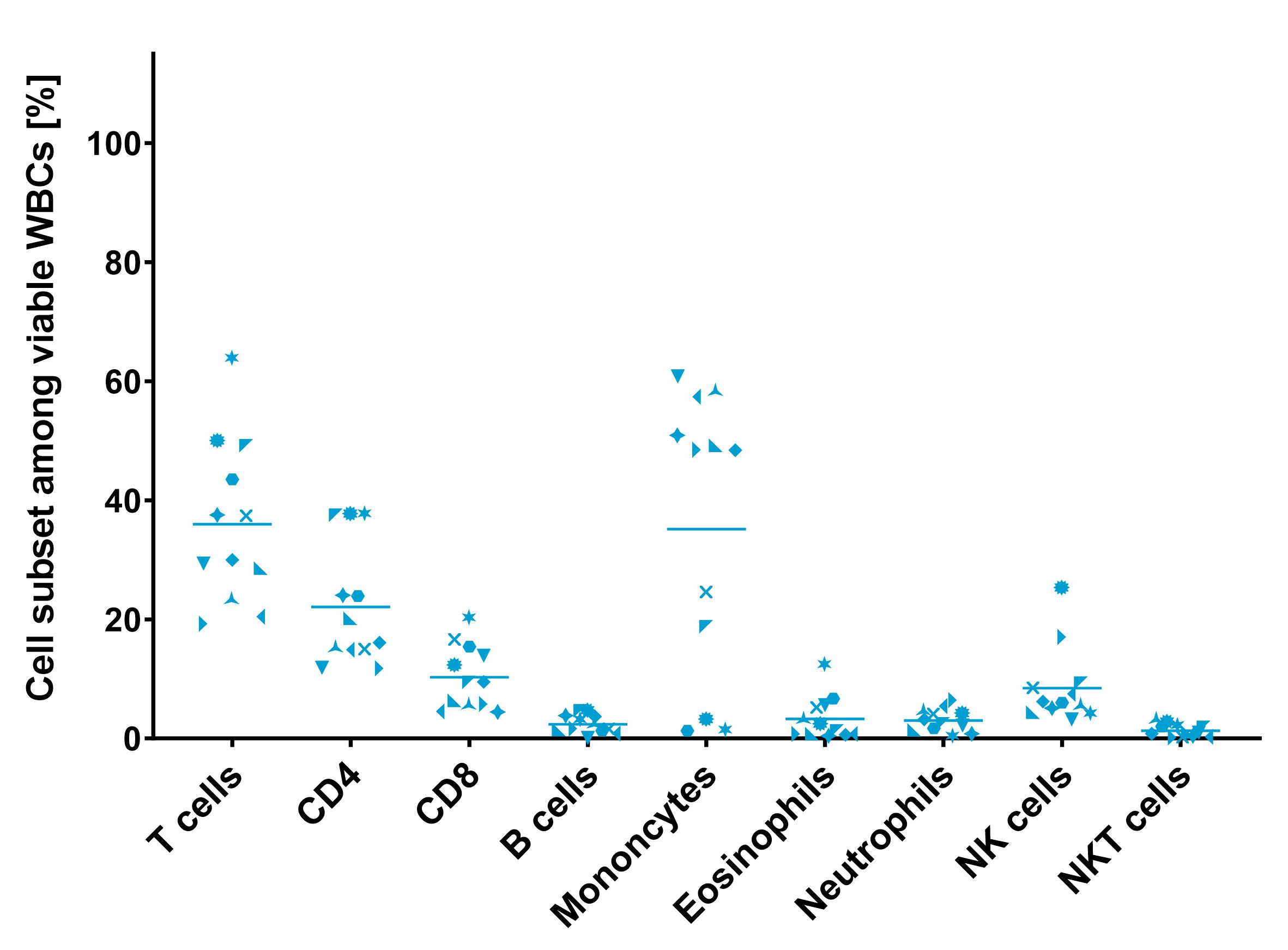

Supplement: Supplementary Figure 3 — Characterization of CD137 enriched virus-specific T cells from LP. The cellular composition in the positive fraction was evaluated after enrichment using flow cytometry analysis (n=12). Line marks the mean. [file Image3.jpeg]

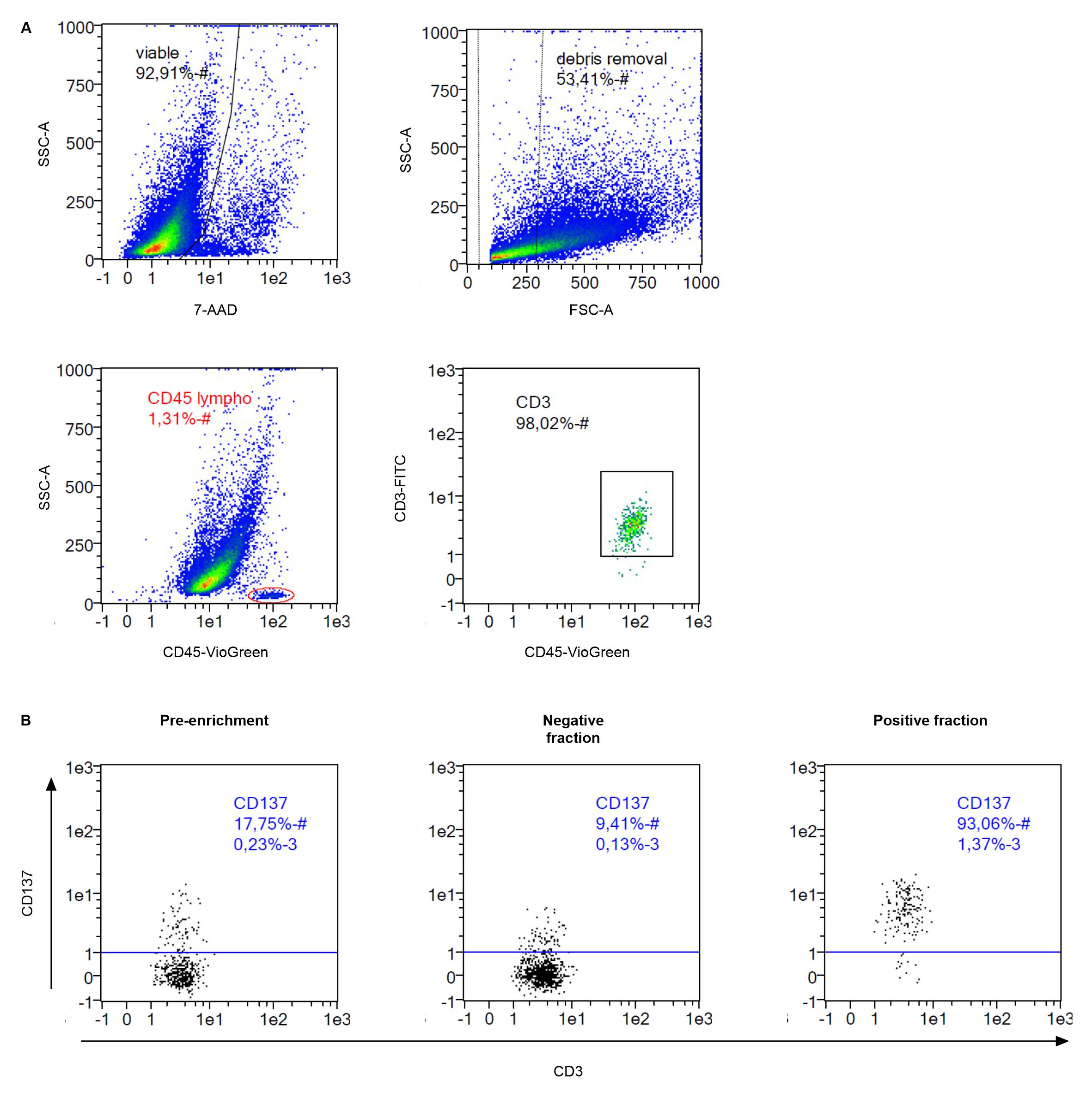

Supplement: Supplementary Figure 4 — Representative flow cytometry analysis of the CD137 expression of a tumor digest sample in the starting material (pre-enrichment), negative and positive fraction after enrichment. (A) Gating strategy for the analysis of the CD137 enrichment performance: Dead and apoptotic cells are excluded by a gate around viable 7-AAD negative cells. Cells are separated from debris via forward scatter (FSC) and side scatter (SSC). Lymphocytes are defined as CD45+ SSClow cells and further gated for CD3+ to identify all T cells. The frequency of CD137+ cells is determined within gated viable CD3+ T cells. (B) Example of CD137-enrichment performance. CD137 frequency pre-enrichment, in the positive and in the negative fraction of the same donor. [file Image4.jpeg]

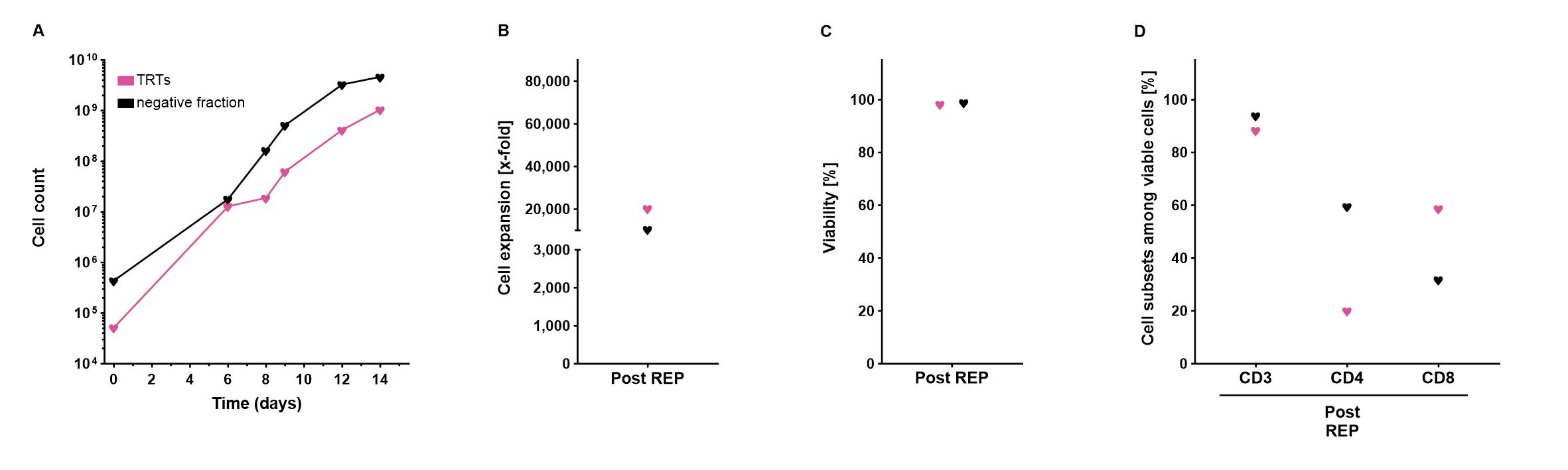

Supplement: Supplementary Figure 5 — Automated expansion of the positive (TRTs) and negative fraction from the CD137 enrichment of the same donor. T cell expansion, viability and the cellular composition of expanded cells was evaluated at the end of the culture using flow cytometry analysis. [file Image5.jpeg]

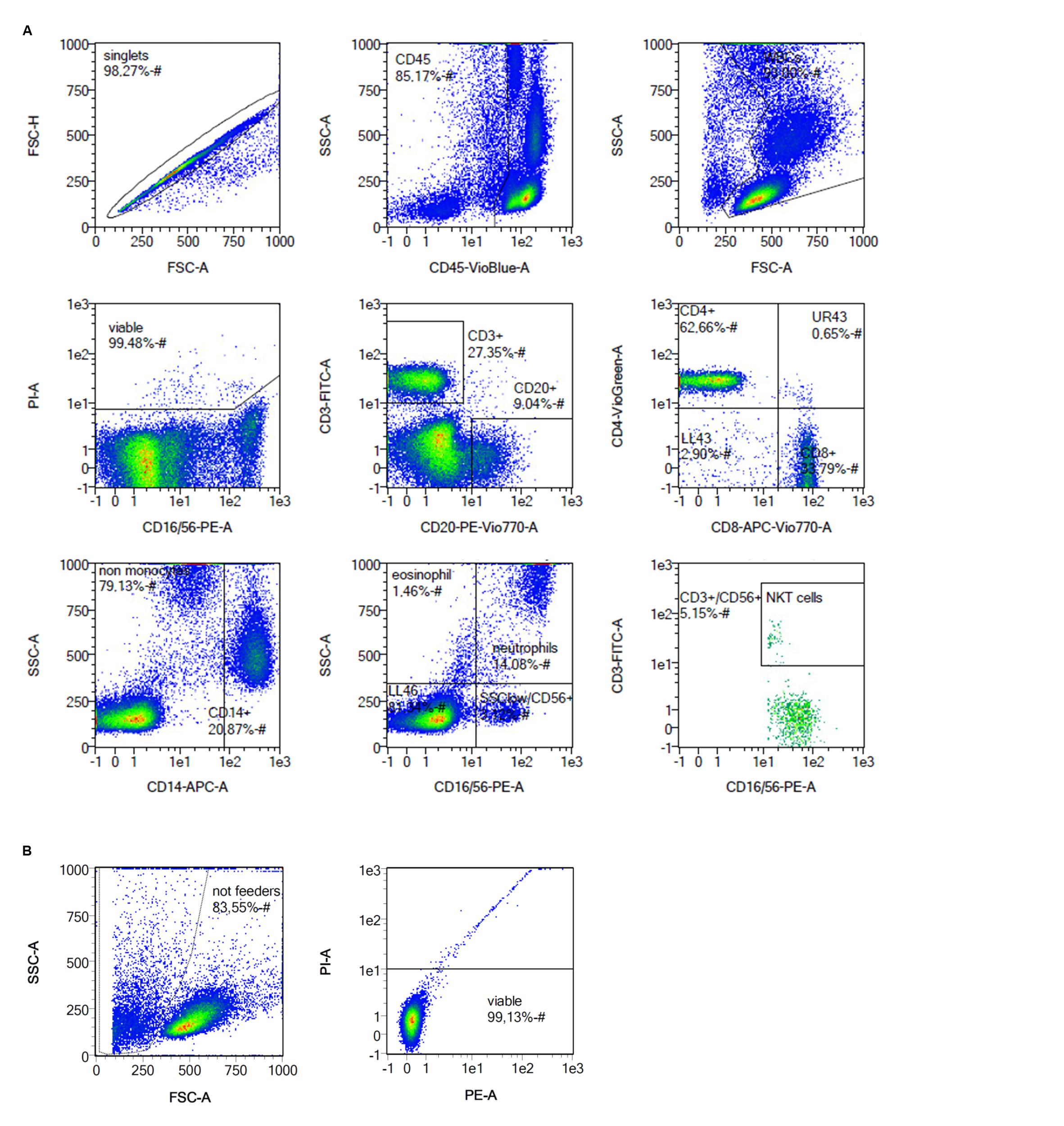

Supplement: Supplementary Figure 6 — Representative gating strategy for the determination of cell counts, viability and cellular composition using flow cytometry analysis. (A) The 8-Color Immunophenotyping Kit is used for viable T cell counts, T cell viability and cellular composition during stimulation and enrichment (exemplary flow data of an LP sample used for enrichment of virus-specific T cells): For elimination of doublets, a gate is set around single cells in forward scatter area (FSC-A) versus forward scatter height (FSC-H). To identify the major circulating blood cell types, CD45 is used to target all leukocytes. These cells are further separated from debris via forward scatter (FSC) and side scatter (SSC). Dead and apoptotic cells are excluded by a gate around viable 7-AAD- cells. B cells are defined as CD20+ while CD3 is used to identify T cells. The T cells are further divided into CD4+ and CD8+ T cells. Monocytes are identified based on their CD14 expression. Among the non-monocyte population, cells are separated into CD16+/SSChigh neutrophils, CD16–/SSChigh eosinophils as well as a CD16-/dim/SSClow population. The CD16-/dim/SSClow population is subdivided based on CD3 and CD56 to distinguish the CD56+ NK cells and a CD3+CD56+ NK T cell population. (B) Samples stained with Propidium Iodide are used for viable cell counts and viability during the expansion (exemplary flow data of expanded yTILs on day 14): Cells are separated from debris via forward scatter (FSC) and side scatter (SSC). Dead and apoptotic cells are excluded by a gate around viable PI- cells. [file Image6.jpeg]
